# Hepatocellular Carcinoma Arising in Non-Cirrhotic Haemochromatosis

**DOI:** 10.1155/1995/46986

**Published:** 1995

**Authors:** N. P. Thompson, G. Stansby, M. Jarmulowicz, K. E. Hobbs, N. McIntyre

**Affiliations:** Hepato-Biliary and Liver Transplantation Unit Royal Free Hospital Pond Street Hampstead London NW3 UK

## Abstract

Hepatocellular carcinoma arising in a patient with genetic haemachromatosis, without cirrhosis, has only been described once previously. We present a patient with a 15 year history of genetic haemachromatosis who underwent resection of a hepatocellular carcinoma in a liver with normal architecture.


*HPB Surgery*, 1995, Vol. 8, pp. 163–166
Reprints available directly from the publisher
Photocopying permitted by license only

© 1995 Harwood Academic Publishers GmbH
Printed in Malaysia

### Hepatocellular Carcinoma Arising in Non-Cirrhotic Haemochromatosis

N. P. THOMPSON, G. STANSBY, M. JARMULOWICZ, K. E. HOBBS and N. McINTYRE

Hepato-Biliary and Liver Transplantation Unit, Royal Free Hospital, Pond Street, Hampstead, London NW3

Hepatocellular carcinoma arising in a patient with genetic haemachromatosis, without cirrhosis, has only been described once previously. We present a patient with a 15 year history of genetic haemachromatosis who underwent resection of a hepatocellular carcinoma in a liver with normal architecture.

KEY WORDS: Hepatocellular carcinoma haemochromatosis

### INTRODUCTION

Hepatocellular carcinoma (HCC) is a well known complication of genetic haemochromatosis (GH), the estimated relative risk being approximately 220[1]. It is widely stated that HCC develops only in patients with cirrhosis in GH[2,3] and most recently "there is no published report of the occurence of liver cancer in a noncirrhotic patient with haemochromatosis"[4]. There are two case reports of HCC after reversal of cirrhosis with venesection[5,6], and one report of HCC developing in two patients with fibrosis but no cirrhosis[7]. We present details of a patient with GH in whom an HCC developed in a liver with normal architecture.

### CASE REPORT

The patient was 72 year old man when he was referred to the Royal Free Hospital. Haemochromatosis had been diagnosed 15 years previously when he presented with a symmetrical small joint polyarthralgia and pigmentation. There was no relevant past history; he had no risk factors for viral hepatitis and drank little alcohol. Investigations at that time revealed a markedly elevated ferritin and serum iron. Liver biopsy showed grade 3 siderosis, a minor degree of fibrosis and a moderate chronic inflammatory cell infiltrate in the portal tracts which was thought to be unrelated to GH. He was treated with regular venesection, initially fortnightly and then monthly. A repeat biopsy one year later showed resolution of the inflammation and less siderosis. Thereafter he remained generally well but developed non-insulin dependent diabetes.

He was followed up regularly with liver function tests and measurements of alphafetoprotein (AFP) which were consistently normal until 1992, when his liver function tests and AFP were noted to have become abnormal over a six month interval.

Examination revealed a few spider naevi and a palpable liver. Subsequent investigations at the Royal Free Hospital included alkaline phosphatase 192 iu/l (normal 35–130), aspartate transaminase 80 iu/l (5–40), alanine transaminase 30 iu/l (5–40) and AFP of 89,200 iu/l (0–10). Serum iron was 17 umol/l (11–36), iron saturation 47% (20–40) and ferritin was raised at 442 ug/l (39–340). CT scan of the abdomen revealed a well demarcated lesion in the right lobe of the liver which, with the markedly raised AFP, was considered diagnostic of an HCC. Serological markers for hepatitis B and C viruses were negative. Bone scan and chest CT scan revealed no evidence of metastatic disease. A biopsy of the left lobe of the liver revealed no evidence of cirrhosis but a considerable amount of iron within hepatocytes. Previous liver biopsies were reviewed at this hospital.

The patient was treated initially by targeted chemotherapy with lipiodol and epirubicin administered angiographically via the hepatic artery; he subsequently underwent a right partial hepatectomy. Histology of the resected specimen revealed a moderately differentiated HCC arising in a liver with normal architecture (Figs. 1 and 2). There was no significant portal inflammation and no fibrosis; however, there was patchy but extensive grade 3 siderosis (Fig 3). The resection margins were free of

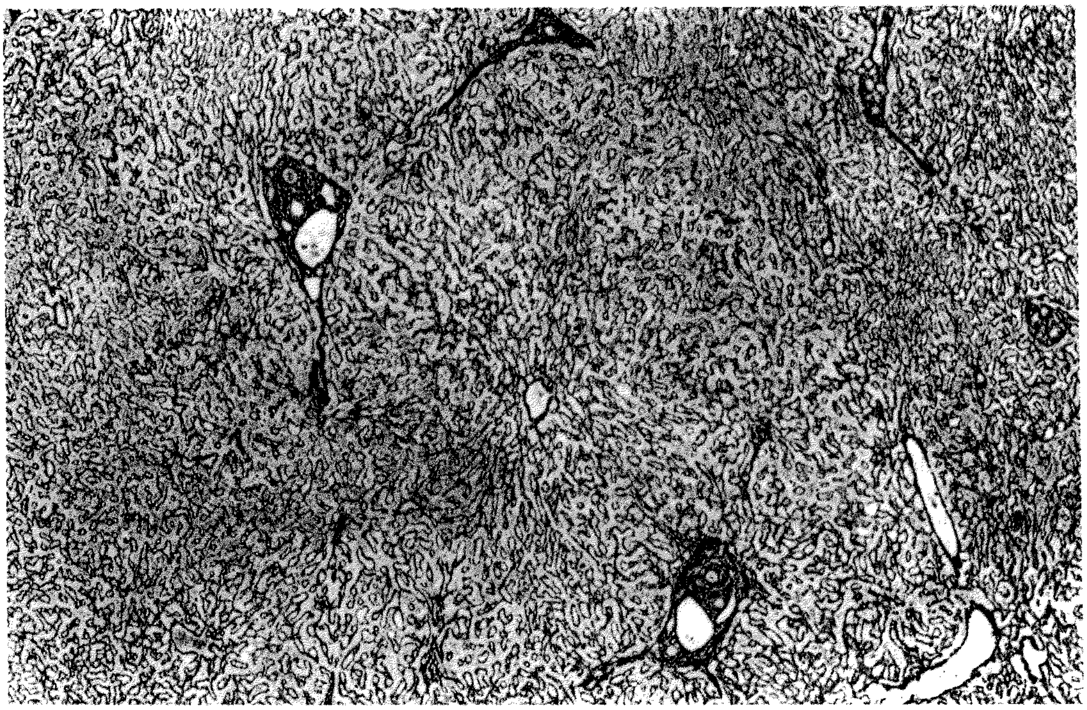

**Figure 1** Gordon and Sweet's Reticulin stain. Shows normal architecture of non-neoplastic liver. Magnification × 40

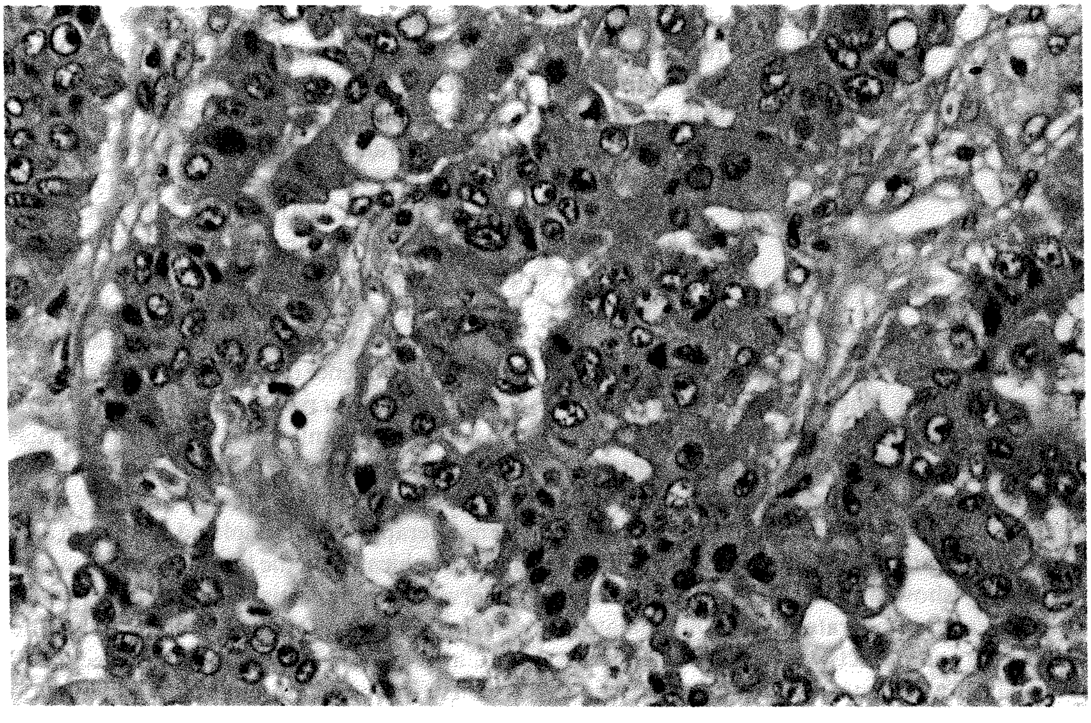

**Figure 2** Haematoxylin and Eosin stain. Hepatocellular carcinoma. Magnification × 400

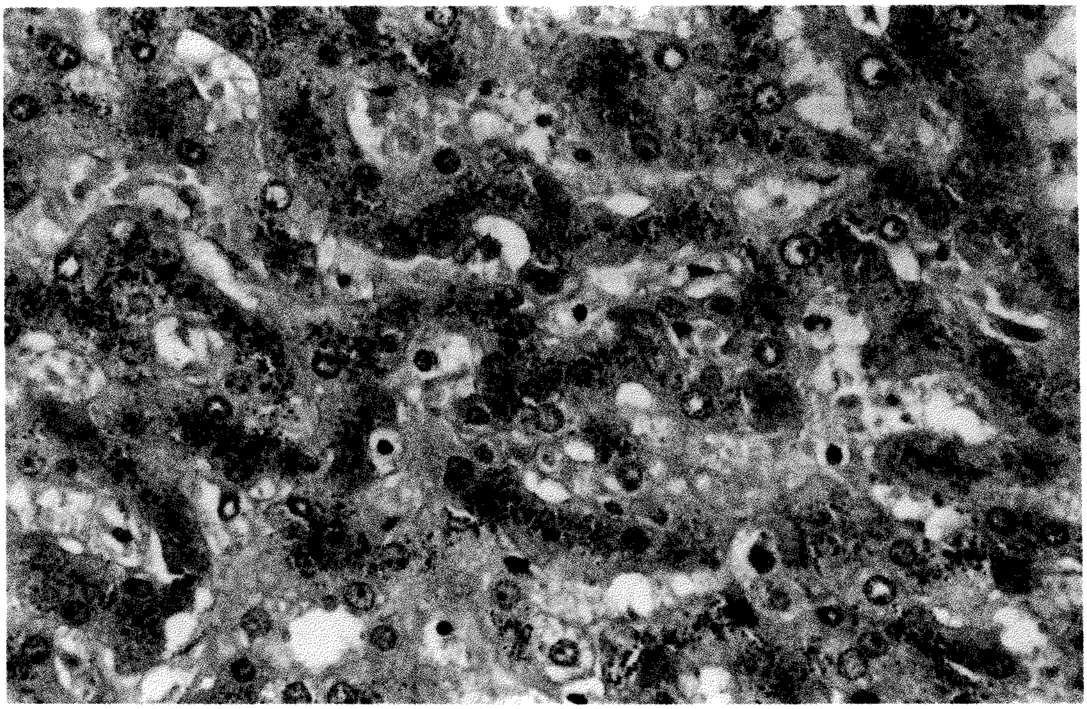

**Figure 3** Perls Prussian blue stain. Shows grade 3 siderosis in non-neoplastic liver. Magnification × 400

tumour. He had an uneventful post-operative course and remains well 9 months later. A high AFP of 3,035 iu/l suggests tumour recurrence although there is no clinical or radiological evidence for this.

### COMMENT

This case demonstrates that hepatocellular carcinoma may arise in the liver of a patient with GH in the absence of cirrhosis; as the patient had a surgical resection the question of sampling error does not arise. Tumour surveillance, therefore, is advisable in patients with GH without cirrhosis as tumours may arise, though very rarely. There were no other identifiable aetiological factors which could explain the HCC.

The pathophysiology of iron overload is incompletely understood. Iron is thought to cause toxicity through its ability to form highly reactive free radicals which cause lipid peroxidation affecting particularly mitochondrial and microsomal membrane lipids[8]. Ensuing lysosomal instability is thought to be fundamental in producing hepatocyte toxicity. Chronic liver cell damage itself, regardless of aetiology, produces a proliferative response which as a result of increased cell growth fosters secondary genetic events. These may be activation of protoonco-genes or inactivation of suppressor genes, thus programming the cell for unrestrained growth[9]. Iron itself, again possibly by peroxidation, has been shown to be directly mutagenic[10]. Animal studies with woodchucks infected with Woodchuck Hepatitis Virus have shown that HCC may develop in the presence active inflammation without the development of cirrhosis[11]. It is therefore not surprising that an HCC arose in a condition known to cause chronic liver damage but without development of cirrhosis; such an event is well recognised in chronic Hepatitis B infection.

This case also suggests that fibrosis may regress, as reported by Blumberg *et al.*,[5] despite incomplete removal of excess iron stores; however, the original amount of fibrosis in 1977 was minor. The portal tract inflammation in the first biopsy, in 1977, is unexplained but its resolution by the following year and absence in the partial hepatectomy specimen makes it unlikely that it is relevant to the aetiology of the HCC.

### REFERENCES

1. Niederan, C., Fischer, R., Sonnenberg, A., Stremmel, W., Trampisch, H. T., Strohmeyer, G. (1985) Survival and causes of death in cirrhotic and non-cirrhotic patient with primary haemochromatosis. *NEJM*, **313**, 1256–1262.
2. Okuda, K., Okuda, H. (1991) Primary liver cell carcinoma. In: McIntyre, N., Benhamou, J-P., Bircher, J., Rhodes, J., eds.

*Oxford textbook of clinical hepathology*. Oxford: Oxford University Press, 1025

3. Bothwell, T. H., Charlton, R. W., Motulsky, A. G. (1989) In: Scriver, C. R., Beaudet, A. L., Sly, W. S., Valle, D. eds. *The Metabolic Basis of Inherited Disease*. New York: McGraw-Hill Information Services Co., 1451.
4. Lankiewicz, M., Spivak, J. L. (1993) Hemochromatosis. In: Rustgi, V. K., Van Thiel, D. H. eds. *The liver in Systemic Disease*. New York: Raven Press, 9.
5. Blumberg, R., Chopra, S., Ibrahim, R., Crawford, J., Farraye, F., Zeldis, J., Berman, M. (1988) Primary hepatocellular carcinoma in idiopathic haemochromatosis after reversal of cirrhosis. *Gastroenterology*; **95**, 1399–1402.
6. Sheehan, F., Connolly, C. E., McCarthy, C. F. (1989) Hepatocellular carcinoma in idiopathic haemochromatosis. *Gut*; **30**, 889.
7. Fellows, I. W., Stewart, M., Jeffcoate, W. J., Smith, P. G., Toghill, P. J. (1988) Hepatocellular carcinoma in primary haemochromatosis in the absence of cirrhosis. *Gut*, **29**, 1603–1606.
8. Bonkovsky, H. L. (1991) Iron and the liver. *American Journal of Medical Sciences*, **301**, 32–43.
9. Okuda, K. (1992) Hepatocellular Carcinoma: Recent Progress. *Hepatology*, **15**, 948–963.
10. Loeb, L. A., James, E. A., Waltresdorph, A. M., Klebanoff, S. J. (1988) Mutagenesis by the autoxidation of iron with isolated DNA. *Proc. Nat. Acad. Sci.* USA, **84**, 3918–3922.
11. Popper, H., Roth, L., Purcell, R. H., Tennant, B. C., Gerin, J. L. (1987) Hepatocarcinogenicity of the woodchuck hepatitis virus. *Proc. Natl. Acad. Sci.* USA, **84**, 866–870.

